# Practical Recommendations for the Preoperative Screening and Protective Protocols in Cancer Surgeries During COVID-19: A Systematic Review

**DOI:** 10.3389/fsurg.2021.678700

**Published:** 2021-11-23

**Authors:** Sara Dorri, Fateme Sari, Seyedeh Nahid Seyedhasani, Alireza Atashi, Esmatalsadat Hashemi, Asiie Olfatbakhsh

**Affiliations:** ^1^Department of Management and Health Information Technology, School of Management and Medical Information Sciences, Health Information Technology Research Center, Isfahan University of Medical Sciences, Isfahan, Iran; ^2^Clinical Research Department, Breast Cancer Research Center, Motamed Cancer Institute, ACECR, Tehran, Iran; ^3^Department of Health Information Technology, School of Paramedical Sciences, Torbat Heydariyeh University of Medical Sciences, Torbat Heydariyeh, Iran; ^4^E-Health Department, Virtual School, Tehran University of Medical Sciences, Tehran, Iran

**Keywords:** COVID-19, preoperative, screening, cancer, surgery

## Abstract

**Introduction:** The new coronavirus (COVID-19) has posed many new challenges to the health care and the timing of surgical care. At the beginning of the pandemic many guidelines recommended postponing elective surgical procedures to reallocate resources. As regards, delay in cancer treatment could be effective on cancer progression. The aim of this systematic review was to outline a guideline for preoperative screening before cancer surgeries and protecting health care workers during the pandemic.

**Materials and Methods:** This study was conducted through a search in electronic databases up to August 2020. PubMed, EMBASE, Web of Science, Scopus, Science Direct, and Google Scholar databases were searched without time limitation. The keywords were a combination of preoperative, cancer surgery, COVID-19, and their synonyms.

**Results:** The most commonly used ways to triage preoperatively were telephone pre-assessment for suspicious symptoms and history of contact or travel, 14-day self-isolation, in- hospital queries at admission, temperature monitoring, and isolation in a single room COVID-free ward or physical distancing. Reverse transcription-polymerase chain reaction (RT-PCR) test 24–72 h before operation was recommended commonly, except in inaccessible centers, but non-contrast chest-CT scan is not routinely advised for elective surgeries to salvage medical resources. Recommended personal protective equipment (PPE) for staffs were wearing N95 mask in addition to gown, gloves, eye protection in aerosol-generating procedures (AGPs), and wearing gloves, hats, and disposable surgical masks, practice distancing, and hand hygiene for all staffs. Meanwhile team separation of hospital staffs caring for COVID-19 patients, segregated areas for COVID-19 clean and contact, restriction of visitors and family members, and personal distancing are mostly recommended.

**Conclusion:** We hope this review would be a guidance for triage, preoperative testing, and summarizing safety principles during COVID-19 pandemic alongside with surgical reintegration.

## Introduction

The new coronavirus (COVID-19) was identified in Wuhan, China in 2019. The WHO officially declared the outbreak a pandemic on March 11, 2020 (1). Coronavirus has spread around the world in a short time. Until December 3, 2020, COVID-19 has infected more than 64 million people all over the world, and 1.5 million died from it (2).

This pandemic has posed many new challenges to the health care and social systems, including shortage of resources such as personal protective equipment (PPE), ventilators, intensive care unit (ICU) beds, and blood resources (3). Infection of health care providers was also a major challenge.

In mid-March 2020, recommendations were released by the American College of Surgeons (ACS) (4), the Society of Gynecologic Oncology, and several medical and surgical professional societies to postpone elective surgical interventions (3–6). The document of the Centers for Medicare and Medicaid Services (CMS) provides recommendations to limit those medical services that could be deferred, such as non-emergent, elective treatment, and preventive medical services for patients of all ages (7). The ACS and CMS have categorized most gynecologic cancer cases as semiurgent. However, the ACS further emphasized that if cancer cases are significantly delayed, this could result in significant patient harm (7–9). On the other hand, the association of time interval from cancer diagnosis to definitive cancer surgery with risk of cancer specific outcomes is poorly understood (10).

While some cases could be postponed, the vast majority of the cancer cases have problems. It is important that the decision to cancel or perform a surgical procedure in patients with cancer have medical and legal dilemmas, and the risk of delay and disease progression should be considered. Cancer operation depends on tumor biology, the stage of tumor, and also the waiting list of newly diagnosed patients (11). In some cases, there are some alternative procedures like neoadjuvant systemic therapy. The Department of Surgical Oncology at MD Anderson Cancer Center have traditionally favored neoadjuvant therapy for many solid tumors. After the outbreak of COVID-19, they initiated or continued this treatment sequencing when possible to postpone surgery to beyond the peak of COVID-19 (12). Also there are some reports of hormone neoadjuvant bridging therapy in patients with asymptomatic COVID-19 infection to reduce waiting time during outbreaks and possible cancer progression (13, 14).

Regardless of priority, we know that COVID-19 related infection is most commonly asymptomatic or demonstrated by mild symptoms. Due to the incubation period, the potential of asymptomatic carriers, and the risk of deterioration of COVID-19 intra- and postoperatively, the status of patients should be confirmed before surgery. Preoperative triage during the COVID-19 pandemic is necessary because of the high risk of mortality during the post- or intraoperative period for patients undergoing surgery during the incubation period.

In Iran we had no prior experience to face this challenging situation. In this regards, we conduct a systematic review to outline the preoperative considerations for cancer surgeries during the pandemic of COVID-19 and protocols for protecting health care workers.

## Materials and Methods

### Search Strategy

First, a basic general search was conducted for a series of studies. The databases PubMed, SCOPUS, Science Direct, and Web of Science were searched until August 10, 2020 by searching on all fields. Citations in-process, which are not indexed with MeSH headings, were also searched. The search query consisted of three subqueries that targeted preoperative, cancer surgery, COVID-19, and their synonyms, all three combined with “AND.” Synonyms within a subquery were combined with “OR.” The search was conducted in English. The search strategy was modified and revised for all databases by two medical informatics specialists. A manual search was also performed for retrieving gray literature and the bibliographies of relevant articles.

### Inclusion and Exclusion Criteria

All original papers describing preoperative protocols for cancer surgery in COVID-19 were included and duplicate studies were removed.

Two authors classified the papers by reading their title and abstract (FS, SNSH). In this phase, from all included articles, the studies that are specifically used for preoperative cancer surgery protocols were selected. The results were compared and discussed until a consensus was reached. If needed a paper was read by another reviewer (SD, AO) to reconcile decision on inclusion. References of papers found to be eligible for inclusion were reviewed manually based on their title and abstract (by AA, FS, SNSH). Intended data such as authors name, article title, year of publication, country, journal name, preoperative triage, and PPE were extracted from studies. Studies which were not original or did not mention their protocols were excluded.

**Figure 1:**
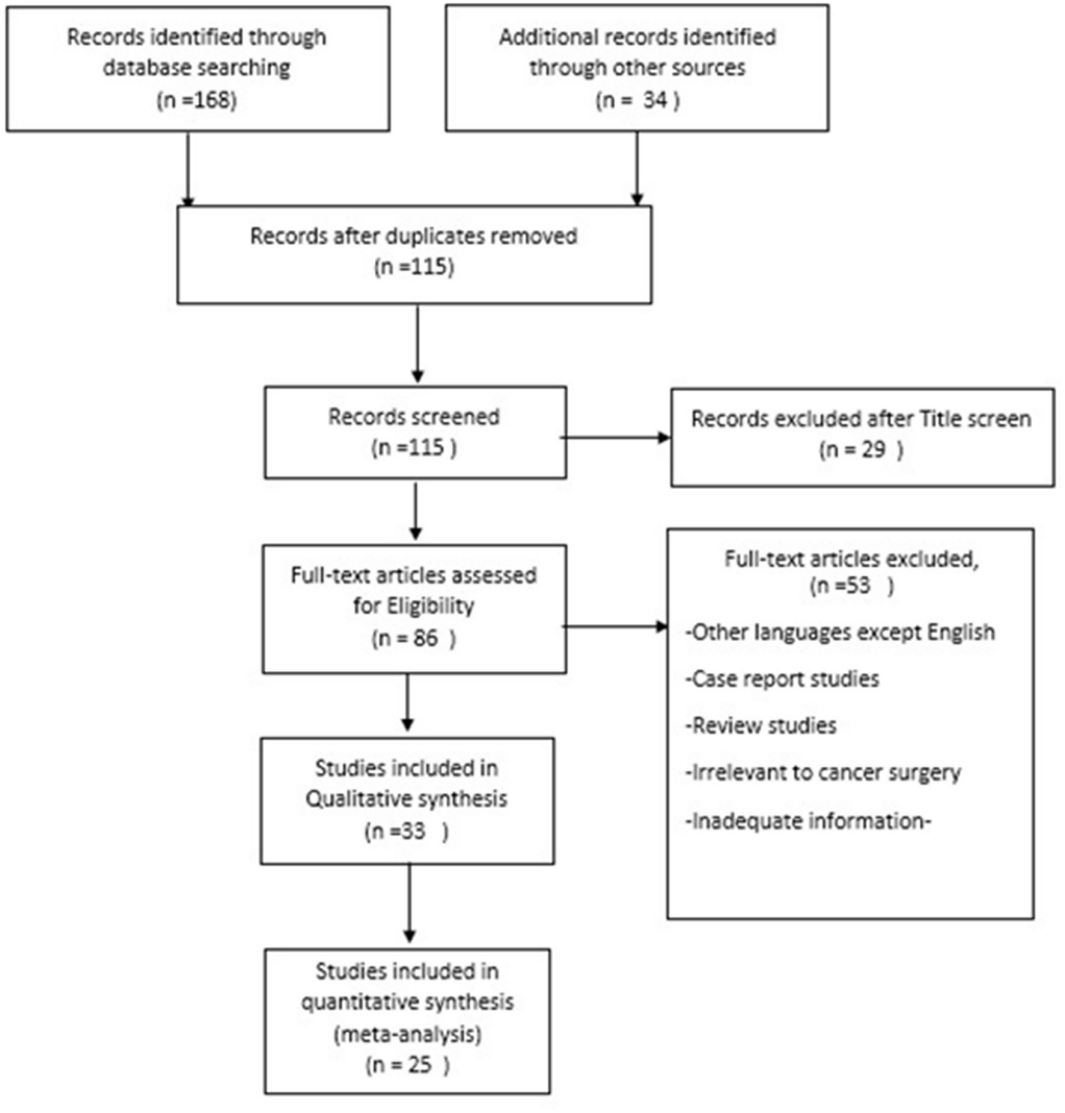
Prisma flowchart of the study.

Prisma flowchart is shown the Figure 1.

## Results

Until the end of searching, 168 articles were retrieved. By hand searching and finding the citations of some articles, 34 articles were added. After removing duplicates, 115 articles remained for further assessment based on title and abstract screening, among them 29 studies were irrelevant and excluded. After full-text assessments, 53 articles were excluded because of the following: other languages except English, irrelevant review studies, irrelevant to cancer surgery, and inadequate information. Finally, 25 articles remained. All evaluation was performed by two authors. Any conflict was solved by a third specialist (surgeon). All articles had acceptable quality and enough explanation about COVID-19 protocols which they had encountered.

About 70% of the articles were Q1 score quality, 19% were Q2, and 11% were Q3. All articles were written in 2020, right after the pandemic. Most articles were about head and neck, gynecology, esophagus, breast, colorectal, and lung cancer surgery. Most of the articles were written in USA (*n* = 6), France (*n* = 4), and China (*n* = 3). Kind of surgery, preoperative triage, and PPE were extracted from these studies. This information is shown in Table 1 and in summary in Table 2.

**Table 1:** The characteristics and the preoperative triage recommendations of the included studies.

	**Title (Ref.)**	**First author**	**Publication year**	**Pre-operative triage recommendations**
**1**	Surgery during the COVID-19 pandemic: a comprehensive overview and perioperative care (15)	Al-Balas M	2020	Preadmission history (respiratory or gastrointestinal symptoms, anosmia, history of recent travel, contact with a person at risk to have the 2019-nCoV infection)
**2**	Perspectives on how to navigate cancer surgery in the breast, head and neck, skin, and soft tissue tumor in limited-resource countries during COVID-19 pandemic (16)	Anwar SL	2020	Temperature monitoring
**3**	Esophageal oncologic surgery in SARS-CoV-2 (COVID-19) emergency (17)	Barbieri L	2020	Questionnaire (presence of fever, cough, shortness of breath, contact with COVID-19 patients, history of traveling)
**4**	Pandemic recovery using a COVID minimal cancer surgery pathway (18)	Boffa DJ	2020	A phone call assessing (fever > 37.5?C, new-onset cough, dyspnea, contact with patient infected or high prevalence areas).
**5**	Head and neck oncological ablation and reconstruction in the COVID-19 era – our experience to date (19)	Butler D	2020	RT-PCR prior to hospitalization
**6**	When to operate, hesitate and re-integrate: society of gynecologic oncology surgical considerations during the COVID-19 pandemic (20)	Fader AN	2020	Self-reporting any symptoms of COVID-19
**7**	French consensus on management of head and neck cancer surgery during COVID-19 pandemic (21)	Fakhry N	2020	If sufficient testing is available, patients should be tested for COVID-19 before go to hospital
**8**	Breast cancer surgery during the COVID-19 Pandemic: an observational clinical study of the breast surgery clinic at Ospedale Policlinico San Martino – Genoa, Italy (22)	Fregatti P	2020	Screened for symptoms/temperature in hospital
**9**	Changing practice patterns in head and neck oncologic surgery in the early COVID-19 era (23)	Kiong KL	2020	Telephone pre-assessment
**10**	Impact of the initial phase of COVID-19 pandemic on surgical oncology services at a tertiary care center in Eastern India (24)	Sultania M	2020	14-day self-isolation
**11**	Optimizing response in surgical systems during and after COVID-19 pandemic: lessons from China and the UK–perspective (25)	Liu Z	2020	COVID-19 PCR swab 48 h pre-operation
**12**	Safe colorectal surgery in the COVID-19 era – a Singapore experience (26)	Tan WJ	2020	Temperature check
**13**	Major head and neck reconstruction during the COVID-19 pandemic: the University of Pittsburgh approach (27)	Ranasinghe V	2020	Chest CT scan on the day of admission in patients requiring level 2 or 3 post-operative care
**14**	Considerations for the surgical management of early stage lung cancer during the COVID-19 pandemic (28)	Merritt RE	2020	Limitation/Elimination of visitors for at least 2 weeks prior to the procedure
**15**	Surgical cancer care in the COVID-19 era: front line views and consensus (29)	Pinheiro RN	2020	Pre-visit phone screen
**16**	COVID-19 and the global impact on colorectal practice and surgery (30)	Nunoo-Mensah JW	2020	PCR-based tests for nasopharyngeal or oropharyngeal secretions
**17**	Surgical activity during the Covid-19 pandemic: results for 112 patients in a French tertiary care center, a quality improvement study (31)	Philouze P	2020	Clinical screening (viral symptoms or potential contact with high risk individuals)
**18**	The impact of preoperative screening system on head and neck cancer surgery during the COVID-19 pandemic: recommendations from the nationwide survey in Japan (32)	Ota I	2020	In settings where RT-PCR testing is not available, and in areas of high prevalence, chest CT (diagnostic purposes among symptomatic patients) may be the only option for preoperative testing
**19**	Surgical outcomes after systematic preoperative severe acute respiratory syndrome coronavirus 2 (SARS-CoV-2) screening (7)	Tilmans G	2020	RT-PCR testing ± chest CT-scan should systematically be performed <24 h before surgery
**20**	Framework for prioritizing head and neck surgery during the COVID-19 pandemic (3)	Topf MC	2020	Telephone triage (7 days before the hospitalization)
**21**	Surgical decision-making and prioritization for cancer patients at the onset of the COVID-19 pandemic: a multidisciplinary approach (33)	Tzeng CWD	2020	In-hospital triage
**22**	Recommendations on management of gynecological malignancies during the COVID-19 pandemic: perspectives from Chinese gynecological oncologists (34)	Wang Y	2020	Patients should use an automatic hand sanitizer dispenser, disposable face, disposable latex-free gloves
**23**	Considerations for head and neck oncology practices during the coronavirus disease 2019 (COVID-19) pandemic: Wuhan and Toronto experience (35)	Wu V	2020	A COVID-19 questionnaire (including health clinical data such as body temperature and any COVID-19 symptoms occurring during the past 2 weeks)
**24**	Clinical characteristics of COVID-19 after gynecologic oncology surgery in three women: a retrospective review of medical records (36)	Yang Sh	2020	Checking body temperature
**25**	Head and neck cancer care in the COVID-19 pandemic: a brief update (8)	Yuen E	2020	Nasopharyngeal (NP) swab and humoral tests for IgM and IgG COVID-19 antibodies only for investigational purpose

**Table 2:** The type of surgeries and the summary of triage recommendations.

	**Authors**	**Country**	**Type of surgery**	**Telephone pre-assessment assessment**	**In hospital assessment history of contact or travel**	**Temperature monitoring**	**14-Day isolation**	**CRP/ESR/CBC**	**RT-PCR prior to hospitalization**	**IgG/IgM**	**Chest CT**	**CXR**	**Isolated ward**
1	Al-Balas M (15)	Jordan	Cancer surgery		✓								
2	Anwar SL (16)	Indonesia	Cancer surgery		✓	✓							
3	Barbieri L (17)	Sweden	Esophageal oncologic	✓					✓		✓		
4	Boffa DJ (18)	Italy	Cancer surgery		✓	✓			✓				
5	Butler D (19)	UK	Head and neck cancer	✓		✓	✓		✓		✓		
6	Fader AN (20)	USA	Gynecologic oncology	✓	✓		✓		✓		✓		
7	Fakhry N (21)	France	Head and neck cancer						✓		✓		
8	Fregatti P (22)	Italy	Breast cancer	✓	✓	✓			✓	✓			✓
9	Kiong KL (23)	USA	Head and neck cancer						✓				
10	Sultania M (24)	India	Oncological		✓	✓			✓				
11	Liu Z (25)	China	All surgery						✓	✓	✓		
12	Tan WJ (26)	Singapore	Digestive		✓						✓	✓	
13	Ranasinghe V (27)	USA	Head and neck cancer	✓					✓				
14	Merritt RE (28)	USA	Head and neck cancer	✓	✓				✓				
15	Pinheiro RN (29)	Brazil	Oncological		✓				✓		✓		✓
16	Nunoo-Mensah JW (30)	UK	Colorectal						✓		✓		✓
17	Philouze P (31)	France	All surgery						✓		✓		✓
18	Ota I (32)	Japan	Head and neck cancer				✓		✓		✓		
19	Tilmans G (7)	France	Digestive		✓				✓		✓		
20	Topf MC (3)	USA	Head and neck		✓				✓				
21	Tzeng CWD (33)	USA	Oncological				✓		✓				
22	Wang Y (34)	China.	Gynecological					✓	✓		✓		
23	Wu V (35)	Canada	Oncological						✓				
24	Yang Sh (36)	China	Gynecological						✓		✓	✓	
25	Yuen E (8)	USA	Oral Oncology		✓				✓				

### Type of Surgery

The categories of studies included seven cancer surgeries, three gynecologic cancer surgeries, eight head and neck and oral cancer surgeries, four digestive surgeries, two all types of surgeries including cancer, and only one article about breast cancer surgery.

### Preadmission Assessment

In 15 articles preadmission process was explained. Six of them had telephone pre-assessment by a breast care nurse who asked them about COVID-19 symptoms and history of contact. Where possible, the preoperative review was completed more than a week ahead of the planned date of surgery and then allowed for 14 days self-isolation (19, 20, 32, 33). Twelve in-hospital assessment included history of contact or travel, and two of them used both (telephone call and in-hospital). Their assessment usually consisted of queries regarding the presence of viral symptoms including fever, cough, myalgia, dyspnea, diarrhea, nausea, and vomiting, and history of contact with high risk individuals, including those in quarantine with active COVID-19. At the time of admission, patients were clinically screened at entry into the institution and any positive screen was evaluated more precisely. Isolation in a single room, COVID-free ward was recommended in four articles (22, 29–31). Temperature monitoring was recommended in five articles and only negative patients were permitted access to the surgery unit (16, 18, 19, 22, 24).

Anesthesiologist consultation to select patients carefully with comorbidities and to discused about the potential COVID-19 related complications during the perioperative period (26). After completing preoperative review, a 14-day self-isolation was recommended in four articles to assist social distancing and to decrease the risk of asymptomatic infection (19, 20, 32, 33).

### Preoperative COVID-19 Testing

Access to reliable testing for the presence of active virus has been a major challenge during this pandemic due to limited testing capacity, reagents, and supplies. Twenty-two of all 25 studies recommended routine preoperative COVID-19 test using real-time reverse transcription-polymerase chain reaction (RT-PCR) with nasopharyngeal or oropharyngeal swab between 24 to 72 h before surgery (Table 2). In one study, a repeated nucleic acid test in 24 h was recommended (25). Samples were obtained in-house or in-hospital. Operation was delayed in patients with a positive test until asymptomatic for 14 days, and then the patient was referred to a structure or a team specialized in the management of COVID-19. Some centers also recommended subsequent documentation of negative RT-PCR testing before proceeding to the operating room (20, 21). Patients who tested negative had a confirmatory telephone triage repeated on the day before hospitalization (22). A recent literature review and pooled analysis of previously published studies on RT-PCR performance from nasopharyngeal specimens demonstrated that the false negative rate of COVID-19 ranged from 20 to 100% depending on the test time since exposure to symptom onset (20). COVID-19 RT-PCR tests appear to be 100% specific (20). Humoral tests for IgM and IgG COVID-19 antibodies were performed only for investigational purpose and did not affect the surgical program in one article (22), and the coronavirus-specific IgM antibody was tested positive in another one in combination with chest-CT (25).

### Chest CT-Scan or Chest-X ray

A non-contrast chest CT scan on the day or 1 day before admission to evaluate for any features consistent with COVID-19 infection was used in some studies (7, 17, 19–21, 25, 26, 29–32, 34, 36). Due to the limited laboratory testing capabilities, the diagnosis in many studies was not based solely on laboratory testing; therefore, a combination with chest-CT was proven to be effective during the outbreak. The sensitivity of chest-CT for COVID-19 was reported between 60 and 97% (7, 17, 25), and some patients had an initial positive chest-CT even before a positive RT-PCR result. Generally, the incubation period for COVID-19 can last 2 weeks or longer considering that CT scan rather than x-ray of the chest plays an essential role in the diagnosis of COVID-19 (36). However chest CT scans was not routinely practical for the preoperative screening of any elective surgery, especially in low-prevalence and low-income countries. This was because of avoiding wastage of medical resources (19, 20, 30, 32). Chest-X-ray was recommended as the alternative preoperative modality for chest-CT scan only in two articles (26, 36).

### Personal Protective Equipment

Personal protective equipment was pointed in 22 of all the articles. The most recommendations were: Wearing mask, usually surgical mask, FFP2 or FFP3. N95 mask was reserved for aerosol-generating procedures (AGPs) like otolaryngology, endoscopy, thoracic surgeries, and for anesthesiologists (20, 21, 26, 29, 33). Also wearing gloves (double-layered in suspicious or unknown cases), gowns, eye protection, face shield, caps, footwear, and meticulous hand hygiene are referred to as standard PPE (3, 15–17, 20–23, 25–30, 32, 34, 35).

Screening symptoms and daily or twice daily temperature monitoring of health-care workers were mentioned in four articles (15, 16, 18, 29). Using minimum number of staff in the operation room (20, 24, 29) and negative pressure or good air exchange in the operating room (8, 20, 26) were also considered. The type of surgery and triage recommendations are summarized in Table 2.

## Discussion

### Overview

This systematic review provided comprehensive considerations for preoperative cancer triage in COVID-19 pandemic. Results revealed that preoperative triage process should start before patient admission till after surgery. Telephone pre-assessment, if possible, asking for suspicious symptoms, history of contact or travel, and if negative, a 14-day self-isolation is one of the best ways for preoperative triage. On the day of admission, it should be continued by in-hospital queries, temperature monitoring, and isolation in a single room COVID-free ward or physical distancing. Reverse transcription-polymerase chain reaction test 24–72 h before operation was recommended commonly, except in unaccessible centers. Non-contrast chest-CT scan on the day or 1 day before admission was recommended only in conditions like limited laboratory testing, but not routinely for elective surgeries to salvage medical resources. Using PPE for staffs are recommended.

While many surgical communities recommended specific guidelines to triage surgical cases for delaying during COVID-19 pandemic, there is scant guidance on how to resume surgical practice in cancer patients. At the beginning of the pandemic, many national and international scientific societies published recommendations to prioritize cancer management strategies, preserve hospital resources for COVID-19, and reduce the risk of cross-infection. But the association between time interval from cancer diagnosis to definitive cancer surgery with the risk of cancer specific outcomes is a challenging problem and creates anxiety in the patient (10). Vanni et al. evaluated the impact of breast cancer screening suspension and treatment delay during the COVID-19 outbreak. In a multicentric study, they retrospectively analyzed data from four Italian breast units. All patients who underwent breast surgery from March 11, 2020 to May 30, 2020 were compared with patients who underwent breast surgery during the same period of the previous year, defined as the prelockdown group. They reported an increase in lymph nodes involvement, and the most significant factor predictive of major advanced N stage was the waiting time on list before surgery. They predicted that in the coming months, an increase in tumor dimensions might occur, and the importance of maintaining breast cancer screening programs and avoiding oncological treatment delay is recommended (37).

In another study, Turaga et al. utilized the National Cancer Database (NCDB) to measure overall survival of all patients undergoing definitive cancer surgery. In this cohort study, from 2004 to 2016, 4,403,437 cancer patients that underwent definitive cancer surgery were included. Patients with head and neck cancers were specifically excluded due to the increased risk of aerosol transmission in their care, making decision making more complex. They found that most cancer surgeries can be safely delayed beyond current wait time for at least 4 weeks without having a significant impact on patient survival or cancer progression. They concluded that these data can be used to assure patients and their family members, and be utilized to build triage systems during a public health emergency (10).

We know that tumor doubling times are not constant. For example, some studies estimated the mean doubling time of breast tumors between 45 and 260 days. This very inaccurate measuring is unhelpful in determining the effect of screening delays on breast cancer survival. However, it is estimated that in 6 months, upto 50% of cases of breast cancer could exhibit a growth of more than the size of a centimeter (38).

Another issue is that in the case of being a candidate for surgery, COVID-19 infection is most commonly asymptomatic or demonstrated by mild symptoms, and because of the potential of asymptomatic carriers and the risk of deterioration of disease intra- and postoperatively, the status of patients should be confirmed before surgery. Yang et al. described the clinical characteristics and outcomes of the patients who were unintentionally scheduled for elective surgeries during the incubation period of COVID-19 infection. In this retrospective cohort study of 34 operative patients with confirmed COVID-19, 15 patients (44.1%) needed ICU care and the mortality rate was 20.5% (36).

A multicentric study, which was funded by the National Institute for Health Research (NIHR), was conducted in patients who had surgery from 235 hospitals in 24 countries and included all surgical patients who were confirmed to have COVID-19 infection. The primary and secondary outcomes were the 30-day mortality rate after surgery and pneumonia, acute respiratory distress syndrome, or accidental ventilation after surgery. Among 1,128 patients undergoing surgery, 294 patients (26.1%) were confirmed to have COVID-19 infection before surgery, their 30-day mortality rate was 23.8%, and pulmonary complications occurred in 51.2% of patients with perioperative COVID-19 infection. They suggested that the increased risks associated with COVID-19 infection should be balanced against the risks of delaying surgery in individual patients (39).

Another important reason to think about treatment delay in addition to the risk of COVID-19 infection is the enormous pressure on health services which emphasize this evaluation. There is a reality that we will face COVID-19 for months, if not years. So reorganization for cancer treatment should be prioritized to maximize the safety of patients, surgeons, and other healthcare professionals during the COVID-19 pandemic.

The referral pathway through the multidisciplinary team (MDT), an individualized plan according to the hospital and regional resources, and to specially introduce anesthetists in this team could help in safely continuing oncologic surgery as long as careful preservation of health care system is taken care of. Anesthesiologists' intervention could evaluate routine comorbidities, risk stratification, and clear the perioperative risks that threaten a patient (19).

The first step is assessing patients if there is any symptom like fever, cough, dyspnea, any contact with infected patient, or travel to high-prevalence areas. This information could be obtained by telephone call or other virtual technologies before admission, if possible. Today, with improvement of electronic health it could be done by web-based applications or telemedicine systems, which could be connected with electronic health records (40–42). Then in-hospital assessment should be done through queries. The patient's body temperature should be detected on admission and in the ward (22). Isolation in a single room, COVID-free ward, or maintaining the correct interpersonal distance is mandatory (22). Clinicians should counsel patients about the risks of cancer surgical delay versus COVID-19 related complications when acquired during the perioperative period or when the patient had a false negative test before admission through a consent form (19, 20).

Diagnostic testing involving identification of COVID-19 using RT-PCR has been a major challenge during this pandemic due to limited resources (20); however, this test was recommended in most studies. The only reasons for limitation are the issues of false negative rate and limited COVID-19 testing availability. Theoretically RT-PCR assays for COVID-19 should be highly sensitive in detecting the presence of viral RNA. However, the limit of detection differs among the various respiratory tract sources. The false negative rate of RT-PCR was reported from 20 to 100% depending on the test time point (5). Care must be taken in interpreting RT-PCR tests for COVID-19 infection, particularly early in the course of infection, when using these results as a basis for removing precautions. COVID-19 RT-PCR tests appear to be 100% specific (20). A single negative test cannot reliably exclude COVID-19, and N95 masks should be worn, when available during AGPs (20). The coronavirus-specific IgM antibody can be tested positive in 3–5 days post-infection (25); however, the COVID-19 serology test usually does not affect the surgical program and has only an investigational purpose (22, 43). Blood samples for blood cell counts and C-reactive protein measurements are encountered as routine measurement, not specifically for COVID-19 triage. Surgery in patients with confirmed COVID-19 should be postponed until proof of virus clearance is obtained and the patient is asymptomatic (44).

Published reports from China during the viral outbreak reported the role of screening computed tomography (CT). One study demonstrated that the sensitivity of chest CT was greater than that of RT-PCR (98 vs. 71%, respectively). Another study demonstrated the sensitivity of CT scan in identifying COVID-19 of 68.4% and specificity of 88% in the population in whom the CT chest was performed as a screening examination (45). Their results supported the use of chest CT for screening for COVD-19 for patients with clinical and epidemiologic features compatible with COVID-19 infection, particularly when RT-PCR testing is negative (46). However as preoperative assessment tool, generalizability of these results is limited (19). In settings where RT-PCR testing is not available, and in areas of high prevalence, chest CT may be the only option for preoperative testing. The American College of Radiology recommends that it need not be performed as a screening test for COVID-19 and rather be reserved for diagnostic purposes among symptomatic patients (47). It is clearly important that imaging facilities are better to be used where there is clear benefit for patients (45, 48). Additionally, the same day discharge can minimize length of hospital stay. Enhancing immunity with some dietary components such as dietary protein, omega-3 fatty acids, and vitamin A can improve the conditions of cancer patients and enhance immunity (49).

After authorization of some COVID-19 vaccines by FDA, the recommendation of the National Comprehensive Cancer Network (NCCN) COVID-19 Vaccination Advisory Committee about vaccination before major surgeries of solid tumors is that vaccination has to be taken at least a few days before the surgery (50). The MSKCC recommendation for patients undergoing cancer-related surgery is a few days or a week or two may be before cancer-related surgery^1^. In patients with completed vaccination dose, the risk of unintended infection before and during surgery would be decreased.

### Personal Protective Equipment

Healthcare workers are at high risk to be affected by virus transmission during surgery in asymptomatic patients with negative COVID-19 tests or those in the incubation period. The risk of transmission is through aerosol and droplets especially during AGPs including bronchoscopy, endotracheal intubation, tracheostomy procedures, cardiopulmonary resuscitation, and diagnostic sputum induction. Wearing N95 mask in addition to droplet PPE (gown, gloves, eye protection) are regarded as minimum protection for all staff members in surgeries involving exposure of upper aero-digestive tracts. The daily assessment of personnel health status and recording body temperature is considered. Routine COVID-19 test is not useful except for workers who directly treat COVID-19 patients and who perform AGPs. All medical staff should perform their clinical tasks wearing gloves, hats, and disposable surgical masks, practice distancing, and hand hygiene correctly (15, 18). Medical leave for all staffs with respiratory symptoms, team separation of hospital staffs caring for COVID-19 patients, as well as segregated areas within the hospital for COVID-19 clean and contact, triage of all hospital visitors (16), restriction of visitors and family members and personal distancing (22) are mostly recommended.

Intraoperative safety that should be taken for all aerosolizing procedures, but not limited to, are the use of N95 respirators, face shields, or goggles for eye protection, gloves, gowns, disposable medical caps, and shoe covers in the operating room. Also quick and safe induction of anesthesia and reducing the number of individuals inside the operating rooms to the minimum required are advised (35, 51). Surgeons should wear the usual cap, gowns, footwear protection, and double gloves. N95 masks (when available) and eye protection are recommended for AGPs; surgical mask with eye protection can be used for non-AGPs (20). Some operating rooms have an average of 15–40 air exchanges per hour to clean air, and that if available could be strongly helpful (26).

## Conclusion

There is a reality that the pandemic will not be short-lived although efforts continue to use widespread effective vaccines to protect health care workers and other people. We know that delay in cancer treatment could be harmful for patients. This review could be an applicable guidance to facilitate setting up services in the managing cancer patients in addition to save clinicians and staff during this challenging time. It can easily be transferred to other surgical specialties to reorganize clinical activities and avoid any impairment of survival of cancer patients.

## Data Availability Statement

The original contributions presented in the study are included in the article/supplementary material, further inquiries can be directed to the corresponding author/s.

## Author Contributions

SD: search—title, abstract, and full text assessment and classify—full text evaluation—manuscript writing. FS and SS: search—title, abstract, and full text assessment and classify. AA: search—evaluation of conflicts. EH: manuscript writing. AO: full text evaluation—major contributor in manuscript writing. All authors read and approved the final manuscript.

## Conflict of Interest

The authors declare that the research was conducted in the absence of any commercial or financial relationships that could be construed as a potential conflict of interest.

## Publisher's Note

All claims expressed in this article are solely those of the authors and do not necessarily represent those of their affiliated organizations, or those of the publisher, the editors and the reviewers. Any product that may be evaluated in this article, or claim that may be made by its manufacturer, is not guaranteed or endorsed by the publisher.

## Footnotes

^1^https://www.asco.org/sites/new-files/covid-19/2021-MSK-COVID19-VACCINE-GUIDELINES.pdf.

## Abbreviations

COVID: corona virus disease
RT-PCR: reverse transcription-polymerase chain reaction
CT: computed tomography
AGPs: aerosol generating procedures
WHO: World Health Organization
PPE: personal protective equipment
ICU: intensive care unit
ACS: American College of Surgeons
SGO: Society of Gynecologic Oncology
CMS: Centers for Medicare and Medicaid Services
IgG: immunoglobulin G
IgM: immunoglobulin M
MDT: multidisciplinary team
RNA: ribonucleic acid
NCCN: National Comprehensive Cancer Network
MSKCC: Memorial Sloan Kettering Cancer Center.
